# The Perceived Risk of Hospitalization in Primary Health Care – The Importance of Multidimensional Assessment

**DOI:** 10.1177/23337214211063030

**Published:** 2022-03-18

**Authors:** Sara Santos, Pedro Mota Veiga, Constança Paúl

**Affiliations:** 1 89239 Abel Salazar Institute of Biomedical Sciences—University of Porto, Portugal; 2 CINTESIS, Faculty of Medicine—University of Porto, Portugal; 3 NECE Research Unit in Business Sciences, 451208 University of Beira Interior, Covilhã, Portugal; 4 Higher School of Education, Polytechnic Institute of Viseu, Portugal

**Keywords:** ageing, risk of hospitalization, primary health care, multidimensional assessment

## Abstract

Ageing has increased the use of health services, with a corresponding rise in avoidable hospitalizations. We aimed to assess and characterize the perceived risk of hospitalization in primary health care (PHC). 118 individuals aged ≥65 years, PHC patients, were assessed using the Community Risk Assessment Instrument by their General Practitioner, who identified their perceived risk of hospitalization, at one year. The instrument is composed of three domains (mental state, daily living activities (ADLs) state and medical state). Multivariate logistic regression was used to identify the best model to predict the risk of hospitalization. Four models were estimated, one for each domain and one with all the variables of the instrument. 58.5% were identified as being at risk of hospitalization. The best predictive models are those that include functionality assessment variables (ADL model and Community Assessment of Risk Instrument model). The model that includes all the variables of three domains presents the best predictive value. Mobility problems (Odds Ratio (OR) 16.18 [CI: 1.63–160.53]), meal preparation (OR 10.93 [CI: 1.59–75.13]), communication (OR 6.91 [CI: 1.37–34.80]) and palliative care (OR 4.84 [CI: 1.14–20.58]) are the best predictors of hospitalization risk. The use of multidimensional assessment tools can allow the timely identification of people at risk, contributing to a reduction in hospitalizations.

## Introduction

Globally, it is estimated that, in 2050, one in every six people will be 65 years old or older (UN, 2020), representing approximately 31% of the European population in 2100 (EUROSTAT, 2019).

The presence of several chronic diseases (Nguyen et al., 2019; Ofori-Asenso et al., 2019; Salive, 2013) is one of the main causes for the loss of healthy years. Advanced age, living alone and dependency in performing daily living activities (ADLs), especially instrumental activities (IADLs), are identified as precursors for the increased risk of hospitalization (Brown et al., 2019; Spector et al., 1987). At the same time, the presence of problems related to the decline in cognitive function is increasingly frequent in hospitalizations (Boustani et al., 2010; Chodosh et al., 2004; Wolf et al., 2019), with these being associated with subsequent adverse outcomes such as increased length of hospital stay, functional decline and mortality (Lucke et al., 2018). However, the change of different biopsychosocial factors (Le Reste et al., 2013), such as the existence of a clinical follow-up, preferably by the same physician, may decrease the risk of hospitalization (Gruneir et al., 2016).

The multimorbidity associated with ageing leads to an increase in the number of medical referrals to different specialities, creating the need for a person-centred service, which oversees and coordinates the necessary health and support services, with primary health care (PHC) and the General Practitioner (GP) being the aggregating elements in this process (American Geriatrics Society Expert Panel on the Care of Older Adults with Multimorbidity, 2012). The GP is the patient’s preferential link with PHC and the Portuguese National Health Service (Moura & Barros, 2018), providing a comprehensive and continuous service throughout their life cycle (WHO & UNICEF, 2018). Regardless of the existing problems in access to health services, especially in rural areas where the number of doctors per 1000 inhabitants is lower than the national average (OCDE, 2019), the high quality of the Portuguese PHC is recognized (Simões et al., 2017).

With an ageing population (PORDATA, 2020a), that lives fewer healthy years than the European average (EUROSTAT, 2021; WHO, 2018) and a high prevalence of multimorbidity (Laires & Perelman, 2018; Rodrigues et al., 2018; Romana et al., 2019), a significant level of stress is expected in the Portuguese PHC (Campos Fernandes, 2020; Gontijo Guerra et al., 2019; Prazeres & Santiago, 2015; WHO, 2016). The need to allocate existing resources efficiently highlights the importance of developing multidisciplinary models that provide an integrated response of health and social care services, ensuring its efficiency and sustainability (DGS, 2017). In 2015, over 80% of hospitalizations that could have been avoided occurred in people over 65 years of age (Rocha et al., 2020). PHC is the ideal place for identifying and referring elderly people at risk (Garrard et al., 2020; Rocha et al., 2020; Rubenstein et al., 1991), with the GP being the privileged counterpart for such identification (Moons et al., 2012; Teixeira et al., 2017). The relationship of proximity and continuity gives the GPs the necessary knowledge to perform a multidimensional assessment of the patient’s health condition and, thus, to assess the potential risk of adverse events (Moons et al., 2012; Teixeira et al., 2017).

The use of short, quick and reliable assessment tools could make the difference in time management and daily work of GP’s, the Community Assessment of Risk Instrument (CARI) being a practical example of such tools (Clarnette et al., 2015). The CARI evaluates the GP’s perceived risk for occurrence of adverse events, based on a multidimensional assessment (Clarnette et al., 2015; O’Caoimh et al., 2012). Thus, using the CARI data provided by the GPs, we aimed to identify the perceived risk of hospitalization in a sample of people aged 65 years or older, as well as to identify the variables and models that best explain this risk in the following 12 months.

## Methology

This study is part of a research project that aims to characterize and identify the needs of people in the area of mental health, aged ≥65 years, users of the PHC in the area of influence of the Regional Health Administration of Norte (ARSN) (Paul et al., 2015), between 2014 and 2016. The study was approved by the Ethics Committee of the ARSN (Opinion no. 6/2014). The research protocol and procedures were developed according to the Declaration of Helsinki. Users of health units not covered by ARS Norte, residents in nursing homes, hospitals and psychiatric institutions, aged <65 years and with no subjective complaints of memory deficit were excluded.

The first phase of the study involved the identification/screening, by the GP, of individuals who could present mental health problems, using the Risk Instrument for Screening in the Community (RISC) (O’Caoimh et al., 2014; Santos et al., 2021). Using the stratified probabilistic sampling method, the study sample was identified and those who agreed to participate were included (informed consent). The research included the application of three protocols, being Protocols A (participant assessment) and C (caregiver assessment) completed by the researcher in an interview with the participant (A) and/or caregiver (C). Protocol B was completed by the GP, who had to assess all his patients included in the study (Alves et al., 2019). In the present study, in addition to the sociodemographic data, we will only explore data regarding the GP’s assessment included in Protocol B, which include the CARI and the identification of the number of diseases of each individual, with the aim of characterize and assess the perceived risk of the occurrence of hospitalization in the following 12 months.

The CARI (Clarnette et al., 2015; O’Caoimh et al., 2012; O’Caoimh & Molloy, 2012), is an extended version of RISC (O’Caoimh et al., 2015; O’Caoimh et al., 2014; Santos et al., 2021) used in the initial screening. The CARI assesses the perceived risk of institutionalization, hospitalization and death (1 minimum/rare to 5 extreme/certain) within the next 12 months. The evaluation is based on the presence of problems in three domains: mental state, ADLs and medical state (yes/no). Each domain is composed of several items and the GP identifies the presence of a concern in one each of them, assessing its severity (mild/moderate/severe) and the ability of the care network to manage the situation (can manage/carer strain/some gaps/cannot manage/absence). Mental state is composed of 7 items (e.g. cognition and insight), ADLs are composed of 15 items, of which 8 are basics ADLs (e.g. transfer and mobility) and 7 are IADLs (e.g. technology use and shopping) and medical state (e.g. chronic medical conditions) (O’Caoimh & Molloy, 2012).

Descriptive statistical analysis was used to characterize the sample (gender, age, years of formal education, marital status, employment situation, living arrangements, support network and number of diseases). For categorical variables, relative frequencies were used. For quantitative variables, the mean was used as a measure of central tendency and the *SD* as a measure of dispersion. The main outcome variables were the presence of concerns in all items of the different domains of CARI and the risk of hospitalization. We used binary logistic regression models because the dependent variable was binary, and the observations were independent of each other. Univariate logistic regression was used to assess the individual impact of the predictor variables on the risk of hospitalization, and the multivariate logistic regression models were used to identify the best predictor model. All variables with significance values less than *p* < .20 were included in the models, the only selection criterion. We estimated four multivariate models: multivariate model adjusted for gender and mental state; multivariate model adjusted for gender and ADLs; multivariate model adjusted for gender and medical state and multivariate model adjusted for gender mental state, ADLs and medical state. Multivariate logistic regressions were estimated. Results are presented as odds ratios along with the 95% CI. The maximum likelihood method was used for the estimation of the various model parameters, and the existence of variables with potential multicollinearity effects was analysed through the variance inflation factors (VIF), with values of less than five. The model validation was performed with the Hosmer–Lemeshow adjustment test. The data obtained were treated with IBM SPSS software version 27.0 (IBM Corporation, New York, USA). A significance level of 5% (*p* ≤ .05) was considered to determine statistically significant associations.

**Figure 1.**
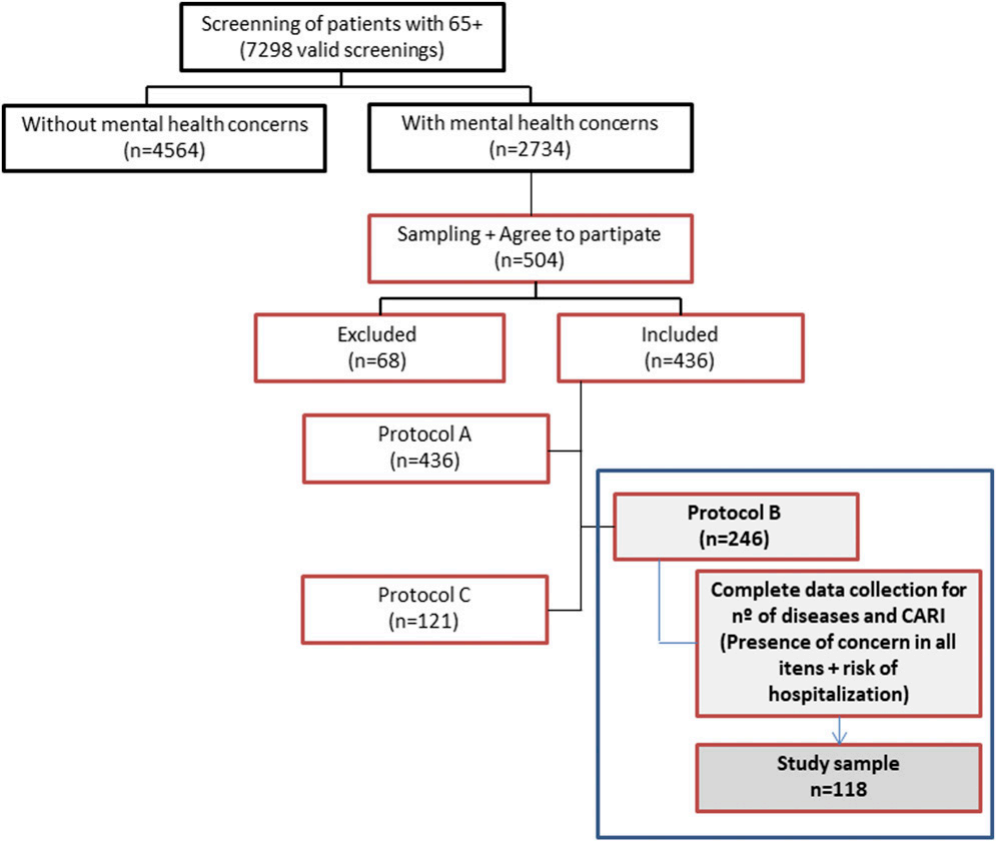
Flowchart of data collection. Protocols A and C were completed by the interviewer with participants (A) and their caregivers (C). Protocol B, which contains the CARI and the disease identification, was completed by the GP of each of the participants.

Although the GPs assessed some variables for 246 individuals, the number of missing data was very high, especially in regard to the CARI items where the n varies from 160 to 230 responses, with the highest number of missing data occurring in the ADL’s domain. This fact led us to analyse only the data of individuals for whom the existence of problems had been identified in all items of the three domains, with the respective assessment of risk of hospitalization as well as the identification of the number of diseases (Figure 1). We only included participants without missing values on outcome criteria in the analysis.

## Results

The sample includes 118 individuals, with a mean age of 77.5 years (*SD*: ±7.39), more than 50% being women (*n* = 66). More than ¼ is illiterate and approximately 70% only attended between 1 and 4 years of formal education. 11.2% live alone and 29.1% receive no support, formal or informal.

Regarding the presence of diseases, 110 have two or more diseases (=93%), reflecting a high level of multimorbidity. 55.9% of the sample has five or more diseases, with woman having a mean number of diseases of 5.23 (*SD* = 2.74) higher than men (4.87 *SD* = 2.55) (Table 1).

**Table 1.** Sample characterization by gender, taking into account age, years of formal education, marital status, employment situation, living arrangements, support network and number of diseases.

		Gender		
		Male	Female	Total
Age	65–74	13.6%	22.9%	43 (36.4%)
	75–87	23.7%	23.7%	56 (47.5%)
n = 118	≥85	6.8%	9.3%	19 (16.1%)
	Mean (SD)	78.13 (6.78)	77.00 (7.85)	77.50 (7.39)
	0	9.3%	17.8%	32 (27.1%)
Formal education	1–4	33.1%	36.4%	82 (69.5%)
n = 118	≥5	1.7%	1.7%	4 (3.4%)
	Single	2.6%	1.7%	5 (4.3%)
Martial Status	Married	33.3%	24.8%	68 (58.1%)
n = 117	Divorced	.9%	1.7%	3 (2.6%)
	Widower (ed)	7.7%	27.4%	41 (35.0%)
Employment situation	Retired	46.4%	53.6%	110 (110%)
n = 110	Employee	.0%	.0%	0 (.0%)
Living arrangements	Alone	4.3%	6.9%	13 (11.2%)
n = 116	With others	39.7%	49.1%	103 (88.8%)
	Only Informal support	17.9%	19.7%	44 (19.6%)
Support	Only Formal support	5.1%	4.3%	11 (9.4%)
n=117	Both	9.4%	14.5%	28 (23.9%)
	None	12%	17.1%	34 (29.1%)
	≤1	3.4%	3.4%	8 (6.8%)
	2	3.4%	5.9%	11 (9.3%)
	3	9.3%	6.8%	19 (16.1%)
	4	4.2%	7.6%	14 (11.9%)
Number of diseases	5	7.6%	10.2%	21 (17.8%)
n = 118	6	5.1%	5.9%	13 (11.0%)
	7	2.5%	4.2%	8 (6.8%)
	≥8	8.5%	11.9%	24 (20.3%)
	Mean (SD)	4.87 (2.55)	5.23 (2.74)	5.07 (2.66)

Values are presented as absolute value (n) and relative percentage (%) or mean and *SD*.

Table 2 presents the results of the CARI. Problems in cognition (80.5%), executive function (72.9%) and anxiety/depression (72.9%) are the most frequent in the mental state domain. The existence of physical aggression and delirium/hallucinations were the least reported (7.6% and 12.7%) with the latest having the highest level of severity in the domain. In the ADLs, the presence of problems in the IADLs occurs between 58.5% and 72% of the sample. We highlight the use of technology (72%) and cleaning the house/laundry (69.5%) as the most frequent. The highest level of severity (severe) is present in shopping (51.3%), food preparation (46.8) and housekeeping/laundry (45.7%). In basic ADLs (BADLs), more than half of the sample presents problems in bathing (59%) and use of stairs/steps (59%). Bowel control, bathing and dressing show greater severity. In the medical state, the main problems are associated to presence of chronic medical conditions (94%) and gait/falls (59.3%). In this domain, the severity of the problems identified is light in more than 60% of the situations, except for the chronic medical conditions, palliative care and swallowing.

**Table 2.** GP’s perception about patients’ problems and the ability of caregiver network to manage (CARI).

	Concern presence n = 118		Mild	Severity moderate	Severe	Caregiver network ability to manage n (%)					
	n (%)	N	n (%)	n (%)	n (%)	n	Manage	Carer strain	Some gaps	Cannot manage	Absence/liability
Mental state						78	36 (46.2)	30 (38.5)	8 (10.3)	3 (3.8)	1 (1.3)
Cognition	95 (80.5)	94	50 (53.2)	23 (24.5)	21 (22.3)						
Insight and executive function	86 (72.9)	85	48 (56.5)	29 (34.1)	8 (9.4)						
Agitation (restlessness)	31 (26.3)	31	9 (29.0)	17 (54.8)	5 (16.1)						
Aggression (physical)	9 (7.6)	9	4 (44.4)	4 (44.4)	1 (11.1)						
Risky behaviours^a^	31 (26.3)	31	9 (29.0)	18 (58.1)	4 (12.9)						
Anxiety/depression	86 (72.9)	85	42 (49.4)	28 (32.9)	15 (17.6)						
Delusion/hallucination	15 (12.7)	15	4 (26.7)	6 (40.0)	5 (33.3)						
ADLs						70	28 (40.0)	32 (45.7)	8 (11.4)	2 (2.9)	0 (0)
ADL basics											
Bladder	55 (46.6)	52	26 (50.0)	12 (23.1)	14 (26.9)						
Bowel	28 (23.7)	22	4 (18.2)	8 (36.4)	10 (45.5)						
Transfer	47 (39.8)	46	25 (54.3)	11 (23.9)	10 (21.7)						
Mobility	57 (48.3)	56	37 (66.1)	10 (17.9)	9 (16.1)						
Dressing	56 (47.5)	55	28 (50.9)	9 (16.4)	18 (32.7)						
Bathing	68 (57.6)	66	29 (43.9)	15 (22.7)	22 (33.3)						
Stairs/steps	67 (56.8)	65	32 (49.2)	15 (23.1)	18 (27.7)						
Feeding	55 (46.6)	54	30 (55.6)	15 (27.8)	9 (16.7)						
ADL’s instrumental											
Technology use	85 (72.0)	85	39 (45.9)	18 (21.2)	28 (32.9)						
Shopping	78 (66.1)	78	22 (28.2)	16 (20.5)	40 (51.3)						
Food preparation	77 (65.3)	77	28 (36.4)	13 (16.9)	36 (46.8)						
Housekeeping/laundry	82 (69.5)	81	23 (28.4)	21 (25.9)	37 (45.7)						
Transportation^b^	79 (66.9)	78	18 (23.1)	40 (51.3)	20 (25.6)						
Medications	76 (64.4)	76	35 (46.1)	24 (31.6)	17 (22.4)						
Finances	69 (58.5)	69	20 (29.0)	19 (27.5)	30 (43.5)						
Medical state						76	32 (42.1)	34 (44.7)	8 (10.5)	2 (2.6)	0
Chronic medical condition (s)	109 (92.4)	106	30 (28.3)	42 (39.6)	34 (32.1)						
Palliative care^c^	31 (26.3)	31	15 (48.4)	15 (48.4)	1 (3.2)						
Hearing	40 (33.9)	37	28 (75.7)	7 (18.9)	2 (5.4)						
Vision	52 (44.1)	51	39 (76.5)	9 (17.6)	3 (5.9)						
Communication	36 (30.5)	35	27 (77.1)	3 (8.6)	5 (14.3)						
Swallow	10 (8.5)	10	4 (40.0)	2 (20.0)	4 (40.0)						
Nutrition	19 (16.1)	19	13 (68.4)	3 (15.8)	3 (15.8)						
Gait/falls	70 (59.3)	68	45 (66.2)	14 (20.6)	9 (13.2)						
Environment/socioeconomics	34 (28.8)	34	28 (82.4)	6 (17.6)	0 (.0)						
Global risk score	Minimal Rare	Low Unlikely	Moderate Possible	High Likely	Extreme Certain						
	n%	n%	n%	n%	n%						
Institutionalization (n = 117)	34 (29.1)	30 (25.6)	32 (27.4)	18 (15.4)	3 (2.6)						
Hospitalization (n = 118)	24 (20.3)	25 (21.2)	50 (42.4)	14 (11.9)	5 (4.2)						
Death (n = 118)	27 (22.9)	35 (29.7)	43 (36.4)	13 (11)	0 (.0)						

^a^Including self-neglect.

^b^Not referring to driving ability.

^c^Symptoms/palliative care aspects (e.g. pain).

The care network presents major constrains in managing the problems identified in the ADLs, where 45.7% do it with strain, 11.4% with failures and 2.9% cannot manage. The risk of occurrence of institutionalization, hospitalization or death at 1 year was assessed on five levels. Considering that those who were assessed as being at a moderate, high or extreme risk as being at risk, and those assessed at minimal or low risk as not being at risk, according to their GPs, 45.4% of individuals are at risk of being institutionalized, 58.5% hospitalized and 47.4% of dying, within the following 12 months.

The risk of hospitalization is identified in more than half of the sample; thus, it is important to understand which elements/variables better explain the perceived risk of hospitalization assessed by GP’s. As the CARI is composed of three domains, we tested one model for each domain: mental state model, ADL model and medical state model (they included only the items belonging to each domain) and one that included all CARI items (Table 3). All independent variables included in the estimated models presented VIF values lower than 5, suggesting the inexistence of multicollinearity between these variables.

**Table 3.** Extent to which the variables gender, age, number of illnesses and the CARI items explain the GP's perceived risk of hospitalization. Predictive models per domain (mental state, ADL state and medical state) and with all items of CARI (inclusion of variables with *p* < .20). *n* = 118.

			Univariate			Mental state domain model^a^			ADL state domain model^b^			Medical state domain model^c^			CARI model^d^		
			OR	CI 95%	*p*	OR	CI 95%	*p*	OR	CI 95%	*p*	OR	CI 95%	*p*	OR	CI 95%	*p*
Age			1.09	(1.03−1.15)	.004*	1.07	(1.01−1.13)	.027*	1.01	(.94−1.09)	.791	1.04	(.98−1.11)	.225	1.03	(.93−1.14)	.559
Gender−Female			1.06	(.51−2.21)	.878												
N^2^ of disease			1.30	(1.11−1.53)	.001*	1.19	(.99−1.42)	.057	1.29	(1.04−1.61)	.023*	1.25	(1.02−1.53)	.030*	1.33	(.99−1.77)	.051
CARI																	
		Cogenition	5.58	(2.00−15.52)	.001*	5.93	(.86−40.95)	.071							12.97	(.86−196.18)	.065
Mental State		Insight and executive function	2.73	(1.19−6.28)	.018*	.55	(.10−2.97)	.486							.09	(.01−1.07)	.057
		Agitation (restlessness)	1.71	(.72−4.05)	.225												
		Aggression (physical)	2.65	(.53−13.36)	.237												
		Risky behaviours^e^	2.08	(.86−5.03)	.104	1.54	(.58−4.11)	.384							2.28	(.46−11.38)	.314
		Anxiety/depression	1.35	(.60−3.06)	.473												
		Delusion/hallucination	2.13	(.64−7.15)	.219												
ADLs	BADLs	Bladder	3.13	(1.44−6.77)	.004*				1.30	(.38−4.37)	.669				1.04	(.25−4.29)	.957
		Bowel	3.36	(1.24−9.06)	.017*				1.33	(.29−6.19)	.718				1.35	(.22−8.39)	.745
		Transfer	3.17	(1.42−7.09)	.005*				.16	(.03−.91)	.039*				.65	(.01−.57)	.014*
		Mobility	6.91	(2.99−15.96)	<.001*				6.67	(1.08−41.19)	.041*				16.18	(1.63−160.53)	.017*
		Dressing	3.89	(1.77-8.54)	.001*				1.15	(.19−7.07)	.883				1.10	(.15−8.26)	.929
		Bathing	5.33	(2.40−11.83)	<.001*				1.70	(.33−8.79)	.528				2.71	(.43−16.97)	.287
		Stairs/steps	8.28	(3.59−19.08)	<.001*				2.29	(.48−10.94)	.300				1.50	(.25−8.98)	.656
		Feeding	4.31	(1.94−9.56)	<.001*				.89	(.20−3.96)	.878				.94	(.18−4.83)	.938
	IADLs	Technology use	2.97	(1.30−6.81)	.010*				1.37	(.33−5.76)	.667				1.06	(.17−6.55)	.947
		Shopping	4.44	(1.97−10.00)	<.001*				1.17	(.23−5.88)	.849				1.32	(.22−.7.86)	.759
		Food preparation	4.82	(2.14−10.87)	<.001*				5.22	(1.13−24.19)	.035*				10.93	(1.59−75.13)	.015*
		Housekeeping/laundry	3.81	(1.67−8.69)	.001*				.38	(.07−2.21)	.280				.22	(.03−1.76)	.154
		Transportation^f^	4.87	(2.14−11.10)	<.001*				2.87	(.51−16.15)	.231				4.99	(.47−52.76)	.182
		Medications	3.19	(1.46−6.97)	.004*				.37	(.07−1.94)	.243				.39	(.05−2.95)	.359
		Finances	3.05	(1.42−6.54)	.004*				.75	(.14−4.05)	.735				.15	(.01−1.49)	.105
Medical state		Chronic medical condition(s)	-	(.00−)	.999												
		Palliative care^g^	5.32	(1.87−15.13)	.002*							3.26	(1.04−10.23)	.043*	4.84	(1.14−20.58)	.033*
		Hearing	4.43	(1.81−10.83)	.001*							2.31	(.76−6.99)	.139	1.04	(.23−4.71)	.957
		Vision	2.25	(1.05−4.82)	.037*							.71	(.26−1.97)	.516	1.22	(.29−5.12)	.787
		Communication	2.86	(1.20−6.82)	.018*							1.77	(.61−5.12)	.295	6.91	(1.37−34.80)	.019*
		Swallow	7.20	(.88−58.83)	.065							3.31	(.33−33.17)	.308	.59	(.44−7.88)	.687
		Nutrition	1.66	(.59−4.73)	.340												
		Gait/falls	3.27	(1.51−7.05)	.003*							1.53	(.61−3.86)	.370	.83	(.19−3.68)	.808
		Environment/socioeconomics	1.73	(.75−3.99)	.101							1.71	(.43−3.16)	.763	.98	(.24−4.02)	.975

^a^Mental state domain model adjusted age, gender, n^2^ of diseases, mental state items; Hosmer–Lemeshow goodness-of-fit test: *p* = .526 (*p* > .05), $R_{\mathrm{negotation}}^{2}=.257$ .

^b^ADL domain state model adjusted age, gender, n^2^ of diseases, ADL state items; Hosmer–Lemeshow goodness-of-fit test; *p* = .220 (p > .05), $R_{\mathrm{Negotation}}^{2}=.457$ .

^c^Medical state domain model adjusted age, gender, n^2^ of diseases, medical state items; Hosmer–Lemeshow goodness-of-fit test; *p* = .173 (*p* > .05), $R_{\mathrm{Negotation}}^{2}=.347$ .

^d^CARI model adjusted age, gender, n^2^ of diseases, mental state, ADL medical state items; Hosmer–Lemeshow goodness-of fit test; *p* = .351 (*p* > .05), $R_{\mathrm{Negotation}}^{2}=.577\left|n=118\right|*p<\mathrm{.05|}$ .

^e^Including self-neglect.

^f^Not referring to driving ability.

^g^Symptoms/palliative care aspects (e.g. pain).

The risk of hospitalization is predicted by the presence of problems in all ADL items, with an increased likelihood of hospitalization between 2.97 times (Odds Ratio (OR) CI: 1.30–6.81) for technology use and 8.28 times (OR CI: 3.59–19.08) for problems associated with stairs/steps. In medical state, the existence of concern in palliative care item increases this probability by 5.32 times (OR CI: 1.87–15.13), when compared to individuals without these problems. Cognition (OR 5.58 [CI: 2.00–15.52]) and insight and executive function (OR 2.73 [CI: 1.19–6.28]) are the only items that individually significantly increase the risk of hospitalization. Unit increments in age and number of diagnoses increase the likelihood of hospitalization by 9% and 30%, respectively.

With regard to the models tested, we found that all of them are valid, but the ones with the highest explanatory value are the ADL state model (includes only the domain items) and the CARI model (includes all items of the instrument).

In model 1, related to the mental state domain, none of the items assessed is a significant predictor of the risk of hospitalization, with age being the only variable with predictive value, by increasing the risk by 7% for each year. In medical state model (model 2), we identified the need for palliative care (OR 3.26 [CI: 1.04–10.23]) and the increased number of diseases (OR 1.25 [CI: 1.02–1.53]) as significant predictors. In model 3, the items of the ADL state domain were included and the presence of mobility problems (OR 6.67 CI: 1.08–41.19), food preparation, (OR 5.22 [CI: 1.13; 24.19]) and a greater number of diseases (OR 1.29 [CI: 1.63; 160.53]) appear as predictor variables of the perceived risk of hospitalization at 1 year. When we include all CARI items, the perceived risk of hospitalization is explained by the existence of problems in mobility (OR 16.18 [CI: 1.63–160.53]), meal preparation (OR 10.93 [CI: 1.59–75.13]), palliative care (OR 4.84 [CI: 1.14–20.58]) and communication (OR 6.91 [CI: 1.37; 34.80]). The number of diseases presents a *p*-value very close to .050 (*p* = .051), and although it is not a predictor with a significant value, it should be noted that for each accumulated disease there is a 33% increase in the perceived risk of hospitalization.

The CARI model (R^2^_Nagelkerk_ = .577) when compared with models that only include items from each domain explains 12% more of the variability in the risk of hospitalization than the ADL domain model (R^2^_Nagelkerk_ = .457), 23% more than the medical statedomain model (R^2^_Nagelkerk_ = .347) and 32% more than the mental state domain model (R^2^_Nagelkerk_ = .257), being the most appropriate.

## Discussion

This study aims to characterize a sample of people aged 65 and over, from a multidimensional assessment, carried out by GPs in PHC services and to identify the variables and models that influence their perception of the risk of hospitalization within the following 12 months.

Cognition, anxiety/depression and executive function problems are present in more than 70% of the sample. These values may be justified by the fact that the individuals were identified in the screening phase as having problems associated with mental health. Other factors that may affect the results are the high prevalence of illiteracy (27.1%) and low education, the high percentage of people aged ≥80 years (36.4%), this being higher than the 2015 national values (PORDATA, 2020b), the high prevalence of multimorbidity (Barnett et al., 2012; Gunn et al., 2012) and the fact that the sample had its origin in rural areas. The percentage of illiterate people is in line with the values reported for Portugal in 2015 (27.7%) and the number of people who attended five or more years of education (3.4%) is much lower than the national values (21.7%) (PORDATA, 2021). Illiteracy increases the possibility of having cognitive problems by 2.92 times when compared to people with other level of education (Yan et al., 2020). In a study conducted to determine the prevalence of cognitive impairment in a sample of Portuguese older people living in the community, the number of people with cognitive impairment rises with age, which reinforces a relationship between cognitive problems and age (Paúl et al., 2010). In the northern region of Portugal, the area where our sample was collected from, a higher prevalence of cognitive problems was found in rural populations when compared with urban populations (Nunes et al., 2010).

At least 6 in every 10 individuals present IADL’s problems, which is higher than the prevalence in the BADLs. A higher prevalence of problems in the IADLs is usual in elderly people (Spector et al., 1987), a fact that may be even more relevant considering the high prevalence of problems in the mental state and the age of our sample. The increase in age can add up to 4.03 times the difficulty in performing BADLs and IADLs in people aged ≥80 years (Connolly et al., 2017). In addition to age, problems in executive function and depression are associated with the impaired ability to perform ADLs and combined ADLs/IADLs (Connolly et al., 2017), problems that are present in 72.9% of our sample. Besides these factors, the presence of problems in IADLs and ADLs appears associated to the presence of multimorbidity (Ćwirlej-Sozańska et al., 2019), a reality in 93.2% of our sample. The presence of severe cognitive problems leads to limitations in the performance of ADLs and IADLs (Luppa et al., 2010), thus, and taking into account that the ability to perform instrumental activities is one of the greatest predictors of participation in daily life activities (Zisberg et al., 2016), an intervention designed to bridge these gaps may prevent the functional decline and promote autonomy and independence.

With regard to medical state, the presence of problems related to chronic medical conditions occurs in 92.4% of the sample. This fact may be related to the generalized presence of multimorbidity (93.2%), a high mean number of diseases (5.02|DP = 2.66) and the difficulty inherent to their clinical management. The multimorbidity values found in our sample are 14% higher than those identified in Portugal in 2015 (Rodrigues et al., 2018; Romana et al., 2019), a trend that is also seen in the average number of diseases (5.7|*SD* = 2.66 vs. 3.3|*SD* = 2.5) (Rodrigues et al., 2018). In our sample, the number of people with five or more diseases is 55.9%, and these values may be associated with the presence of mental health problems. According to Barnett et al. (2012), the number of physical illnesses is highly associated with the presence of mental health illnesses, with those with five or more diseases having 6.74 times (OR CI: 6.59–6.90) more susceptibility to develop mental illness than those with no illnesses (Barnett et al., 2012). In our study, the prevalence of multimorbidity is higher in women, what corroborated the conclusions of other studies that identify women as having a higher prevalence (Barnett et al., 2012; Laires & Perelman, 2018; Nielsen et al., 2017; Ofori-Asenso et al., 2019; Romana et al., 2019). The analysis of the impact of the different pathologies should be explored in future studies.

The care network presents greater difficulty in managing the problems identified in the ADLs, where 45.7% do it with effort, 11.4% have failures and 2.9% cannot manage. Considering that, approximately 30% of the individuals do not have any type of support, and being these the activities in which the presence of support has greater expression and impact (Alves et al., 2019), this may reflect a scarcity of response of informal support and adequate formal services.

After assessing all the CARI domains, GPs identified 45.4%, 58.5% and 47.7% of individuals as being at risk of institutionalization, hospitalization or death, respectively. Although only an analysis of the risk of hospitalization is performed, the risk of institutionalization and death should be explored in future studies.

The high percentage of people assessed by the GP as being at risk of hospitalization may reflect the perception of an imminently frail population, very susceptible to the occurrence of exacerbations of their multiple chronic diseases (FitzGerald et al., 2014; Kansagara et al., 2011; López-Soto et al., 2019), enhanced by the presence of cognitive problems, identified in more than 80% of the sample.

The risk of hospitalization increases significantly with age and the number of diseases, having this last one a more expressive effect. Our results corroborate other studies where a higher number of diagnoses is associated with a higher probability of being hospitalized (Laires & Perelman, 2018), a higher number of hospitalizations (Palladino et al., 2016) and longer hospital stays (Gruneir et al., 2016; Luben et al., 2020; Ofori-Asenso et al., 2019; Palladino et al., 2016).

The presence of problems in cognition and executive function, in BADLs and IADLs, in palliative care, hearing and vision problems, communication and gait/falls, significantly increase the risk of hospitalization. These results are reinforced by the study of Spector et al. (1987), in which an increased risk of hospitalization at one year was identified when there were problems leading to dependence in the performance of IAVDs (+27%), increasing when there were simultaneous problems in basic and instrumental activities (+37%) (Spector et al., 1987). Besides hospitalization, the corresponding length and the occurrence of deaths during this period is also associated to the presence of these problems in elderly people (Avelino-Silva et al., 2014).

As expected, the existence of problems related to symptoms/palliative care aspects increases, by itself, the perceived risk of hospitalization by 5.32 times. In a sample where 5 out of 10 have five or more chronic diseases, the existence of people with very complex health conditions is a daily reality that increases the need to implement care plans focused on the person, with a multidimensional approach, both at the level of planning and intervention (Gómez-Batiste et al., 2017).

The model that best predicts the risk of hospitalization comprises all CARI items. Thus, the existence of problems in mobility (BADLs), meal preparation (IADLs), palliative care (medical state) and communication (medical state) significantly increased the risk of hospitalization in the following year. These data suggest that an assessment that includes functionality variables, basic and instrumental, make the hospitalization risk assessment more robust than just including mental or medical factors. In clinical practice, these results can be useful for GPs to identify triggering factors for the risk of hospitalization, making it possible for medical referral and timely intervention to minimize them. A better performance of PHC can contribute to a reduction of the number of hospitalizations, the use of hospital services and the financial pressure on health care services (Rocha et al., 2020).

## Conclusions

A multidimensional assessment carried out with instruments such as the CARI can play an important role in identifying people at risk, allowing for an adjustment in the care provided, contributing to a possible reduction in the number of hospitalizations. However, it is important to highlight one of the main limitations identified in our study, which is related to a high number of missing data in the identification of problems in the ADL’s domain. This fact may be related to the difficulty in assessing performance in ADLs in an outpatient setting because, even though the GP’s knowledge about the patient’s health status gives him/her a privileged view, the analysis of different situations regarding daily functionality may not be object of specific attention. Therefore, the inclusion of other health professionals in the analysis and assessment of the elder may overcome this difficulty.
